# Regulatory T Cells Phenotype in Different Clinical Forms of Chagas' Disease

**DOI:** 10.1371/journal.pntd.0000992

**Published:** 2011-05-31

**Authors:** Fernanda Fortes de Araújo, Danielle Marquete Vitelli-Avelar, Andréa Teixeira-Carvalho, Paulo Renato Zuquim Antas, Juliana Assis Silva Gomes, Renato Sathler-Avelar, Manoel Otávio Costa Rocha, Silvana Maria Elói-Santos, Rosa Teixeira Pinho, Rodrigo Correa-Oliveira, Olindo Assis Martins-Filho

**Affiliations:** 1 Laboratório de Imunologia Celular e Molecular, Centro de Pesquisas René Rachou, FIOCRUZ, Belo Horizonte, Brasil; 2 Laboratório de Biomarcadores de Diagnóstico e Monitoração, Centro de Pesquisas René Rachou, FIOCRUZ, Belo Horizonte, Brasil; 3 Laboratório de Imunologia Clínica, Instituto Oswaldo Cruz, FIOCRUZ, Rio de Janeiro, Brasil; 4 Laboratório de Biologia das Interações Celulares, Departamento de Morfologia, Instituto de Ciências Biológicas, UFMG, Belo Horizonte, Brasil; 5 Programa de Pós-graduação em Medicina Tropical, Faculdade de Medicina, UFMG, Belo Horizonte, Brasil; 6 Departamento de Propedêutica complementar, Faculdade de Medicina, UFMG, Belo Horizonte, Brasil; 7 Instituto Nacional de Ciência e Tecnologia em Doenças Tropicais, INCT-DT, Salvador, Brasil; National Institutes of Health, United States of America

## Abstract

CD25^High^ CD4^+^ regulatory T cells (Treg cells) have been described as key players in immune regulation, preventing infection-induced immune pathology and limiting collateral tissue damage caused by vigorous anti-parasite immune response. In this review, we summarize data obtained by the investigation of Treg cells in different clinical forms of Chagas' disease. Ex vivo immunophenotyping of whole blood, as well as after stimulation with *Trypanosoma cruzi* antigens, demonstrated that individuals in the indeterminate (IND) clinical form of the disease have a higher frequency of Treg cells, suggesting that an expansion of those cells could be beneficial, possibly by limiting strong cytotoxic activity and tissue damage. Additional analysis demonstrated an activated status of Treg cells based on low expression of CD62L and high expression of CD40L, CD69, and CD54 by cells from all chagasic patients after *T. cruzi* antigenic stimulation. Moreover, there was an increase in the frequency of the population of Foxp3^+^ CD25^High^CD4^+^ cells that was also IL-10^+^ in the IND group, whereas in the cardiac (CARD) group, there was an increase in the percentage of Foxp3^+^ CD25^High^ CD4^+^ cells that expressed CTLA-4. These data suggest that IL-10 produced by Treg cells is effective in controlling disease development in IND patients. However, in CARD patients, the same regulatory mechanism, mediated by IL-10 and CTLA-4 expression is unlikely to be sufficient to control the progression of the disease. These data suggest that Treg cells may play an important role in controlling the immune response in Chagas' disease and the balance between regulatory and effector T cells may be important for the progression and development of the disease. Additional detailed analysis of the mechanisms on how these cells are activated and exert their function will certainly give insights for the rational design of procedure to achieve the appropriate balance between protection and pathology during parasite infections.

## Introduction

A substantial number of studies have been published on the analysis of the human immune response against the infection by the protozoa *Trypanosoma cruzi*. Those studies have evaluated the putative role of various cell populations, their subsets and cytokines, and their correlation with the regulation of the immune response during *T. cruzi* infection [1]–[13].

Regulatory T cells (Treg cells) have been described as a unique population of CD25^+^ CD4^+^ T cells, a class of cells that regulates innate and adaptive immune responses and has the capacity to control the excessive or misdirected effect of the immune response, including those to pathogens or self-antigens [14]–[19]. In infectious diseases caused by protozoan parasites, a number of publications have focused on the role of Treg cells in patients with Chagas' disease [9], [10], [13], [20], [21].

The purpose of this review is to highlight the progress over the past few years in the investigation of Treg cells in different clinical forms of Chagas' disease. Although new data on the regulatory mechanisms that control diseases continue to accumulate, there is still significant need for further analysis of the various cell populations in *T. cruzi* infection that will allow testing of new hypotheses to elucidate the mechanisms that lead to the development of the different clinical forms of the disease as well as the mechanisms of protection. It is important to mention that the papers cited in this article were selected based on some criteria such as stringency of the papers in relation to the subjects discussed, high quality of papers, and papers indexed in the PubMed database (Box 1).

**Box 1. Methods**

The papers cited in this article were selected based on the following criteria: 1) Stringency of the papers in relation to the subjects discussed. 2) High quality of papers. 3) Papers indexed in the PubMed database.

## Regulatory T Cells in Chagas' Disease

Chagas' disease, or American trypanosomiasis, is a severe infection caused by the haemoflagellate protozoa *T. cruzi*. It is a major public health problem in Latin America, affecting approximately 8 million people in South and Central America [22]. In humans, *T. cruzi* infection usually develops from an oligosymptomatic acute phase to a possibly debilitating chronic phase that can manifest itself in a variety of ways. The majority of patients who progress to the chronic phase remain clinically asymptomatic for many years, with no clinical, radiological, or electrocardiographic manifestations of cardiac or digestive involvement. These conditions characterize the indeterminate clinical form of the disease [23]. Between 30% and 40% of the infected individuals progress to the cardiac (CARD) and/or digestive (DIG) symptomatic disease. It is estimated that 30% of all infected individuals will eventually develop heart disease [23]–[25].

The specific mechanisms associated with the establishment/maintenance of the distinct clinical outcomes of Chagas' disease are undoubtedly extremely complex. Understanding why only a percentage of the infected individuals develop severe manifestations of the disease and why the clinical forms are highly heterogeneous is of major importance not only for the comprehension of the immune mechanisms and clinical forms but, most importantly, for the implementation of adequate therapies and care for these individuals. Several studies have demonstrated that different clinical forms are associated with distinct and complex host–parasite relationships directly involving the immune response [1], [3], [26]–[34]. In fact, it is well accepted that the absence of chagasic pathology is mainly associated with an individual's ability to regulate the anti–*T. cruzi* immune response, which controls persistent parasitism but can also contribute to the inflammatory collateral damage that causes Chagas' disease morbidity.

Our group has, for several years, studied the role of the immune response in the development of the different clinical forms of Chagas' disease. The initial cohort of study subjects was recruited 8 years ago at the Outpatient Referral Center for Chagas Disease of the Hospital das Clínicas, Federal University of Minas Gerais, Brazil. All study participants provided a written informed consent following the guidelines of the Ethics Committee of the Federal University of Minas Gerais. The study protocol complied with the regulations of the Brazilian National Council on Research in Humans and was approved by the Ethics Committees of the Federal University of Minas Gerais under protocol COEP/UFMG-372/04 and the Ethics Committees of the Oswaldo Cruz Foundation under protocol CEP-CPqRR 27/2008.

More recently, we have evaluated the potential role of CD25^High^ CD4^+^ T cells in the control of Chagas' disease pathogenesis [9], [10], [13], [20], [21]. Our previous evaluation of Treg cells throughout the expression of the transcription factor Foxp3 in digestive clinical forms of Chagas' disease showed an increased concentration of Foxp3 T cells in IND group without megacolon compared with those patients with megacolon and non-infected individuals, suggesting that these cells possibly have an important role in the intestinal infection and may represent a mechanism used by patients presenting Chagas' disease to prevent exacerbation of the inflammation and, consequently, avoid megacolon development [21] (Box 2).

**Box 2. Key Learning Points**

- Regulatory T cells have been described as a unique population of CD25^+^ CD4^+^ T cells that regulates innate and adaptive immune responses and has the capacity to control the excessive or misdirected effect of the immune response.
- Regulatory T cells through expression of IL-10 are beneficial to patients in the indeterminate clinical form by maintaining a balance between efficient effector cells that kill the parasites and avoiding the development of tissue immunopathology.
- CD25^High^CD4^+^ T cells use different mechanisms to regulate the immune response during Chagas' disease and the host-parasite interactions may be influenced by the ratio of regulatory/effectors T cells.
- The generation of protection or pathogenic responses in human Chagas' disease is highly influenced by the suggested balance of the complex immune response induced by *T. cruzi.*
- Indeterminate patients have an effective, and possibly transitory, control of the response to the infection mediated by regulatory T cells and effectors cells.

Ex vivo data obtained from patients with indeterminate and cardiac clinical forms of Chagas' disease have shown that there is an increased frequency of CD25^High^ CD4^+^ Treg cells in the peripheral blood of IND patients when compared to CARD and non-infected individuals [9], suggesting that an expansion of CD25^High^ CD4^+^ Treg cells may be beneficial during the chronic phase of the disease, probably by limiting tissue damage, leading to lifelong persistence of the indeterminate clinical form of Chagas' disease. A similar correlation between an increased expansion of this cell population in the peripheral blood and disease progression was previously described for non-progressor, asymptomatic-HIV infected individuals [35].

**Figure 1:**
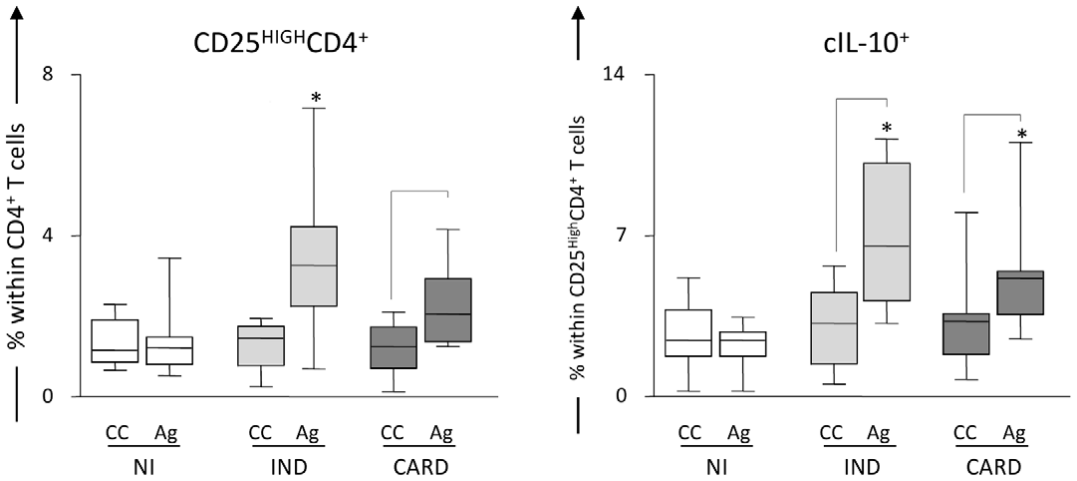
Analysis of IL-10^+^ CD25^High^ CD4^+^ regulatory T cells in the peripheral blood from chagasic patients. Frequency of regulatory T cells and intracytoplasmic IL-10 (cIL-10) levels in CD25^High^ CD4^+^ cells from patients with distinct clinical forms of Chagas' disease (IND, light gray box; CARD, dark gray box) and non-infected individuals (NI, white box) following short-term in vitro stimulation of whole blood samples with *T. cr*u*zi* antigens. Baseline levels of CD25^High^ CD4^+^ and cIL-10^+^ T cells were obtained from control cultures (CC) maintained under the same conditions (22 h incubation at 37°C, CO_2_ humidified incubator). The results are expressed in box plot format for CD25^High^ CD4^+^ (left panels) and cIL-10^+^ T cells (right panels). The box stretches from the lower hinge (defined as the 25^th^ percentile) to the upper hinge (the 75^th^ percentile) and, therefore, contains the middle half of the score in the distribution. The median is shown as a line across the box. Therefore, 1:4 of the distribution is between this line and the bottom or the top of the box. Significant differences are identified by connecting lines for comparisons between CC and Ag, and highlighted the ability of *T. cruzi* antigens to trigger enhanced levels of CD25^High^ CD4^+^ and cIL-10^+^ T cells in both IND and CARD groups. Significant differences between clinical groups are identified by asterisks as compared to NI.

It has been previously described that regulatory T cells can suppress the immune response by producing anti-inflammatory cytokines, such as IL-10 and TGF-β [36]. In an attempt to better characterize these cells in Chagas' disease, we investigated whether CD25^High^ CD4^+^ Treg in the peripheral blood of chagasic patients are IL-10 producers [20]. We observed an increased frequency of cytoplasmic (c) IL-10^+^ CD25^High^ CD4^+^ Treg cells in both IND and CARD individuals after in vitro recall responses induced by exposure to *T. cruzi* antigens (Figure 1). It is interesting to note that our group and others have already described an association between the secretion of IL-10 and the control of immunopathology [3], [4],[9],[34],[37]–[39], contrasting with the observed increase in IL-10^+^ CD25^High^ CD4^+^ Treg cells in CARD. However, it is important to mention that the number of positive cells for this cytokine is higher in IND individuals (unpublished data). The differences mentioned may be explained by increasing evidence that supports the hypothesis that the fine balance of inflammatory and regulatory cytokines derived from distinct T cell sources is the key factor for the resulting immune-mediated mechanisms that drive the disease outcome [3]. In order to confirm if CD25^High^ CD4^+^ T cells are correlated with improved cardiac function and, thus, a possible protective role in Chagas' disease, we performed a correlation analysis between the frequency of these cells and two clinical parameters of cardiac function: left ventricular ejection fraction (LVEF) and left ventricular diastolic diameter (LVDD). These distinct clinical parameters are directly and inversely correlated with better cardiac function, respectively. A significant positive correlation was seen between higher LVEF and higher frequency of CD25^High^ CD4^+^ T cells and a significant negative correlation was seen between lower LVDD and higher frequency of CD25^High^ CD4^+^ T cells. These data suggest that CD25^High^ CD4^+^ T cells display an import immunoregulatory role that leads to maintenance of better cardiac function in IND patients (unpublished data).

Recent data demonstrated that CD4^+^ T cells are the major cell population defining the regulatory profile in IND, whereas monocytes and CD4^+^ T cells determine the inflammatory cytokine pattern in CARD individuals. Interestingly, in vitro stimulation with *T. cruzi* antigen was able to reverse the cytokine balance in IND and CARD groups [11]. In addition to differential immune response, other factors are likely to influence the differential progression of individuals into distinct clinical forms of Chagas' disease, including parasite strain, the size of inoculation, environmental factors, and host genetics, among others [40]–[43].

**Figure 2:**
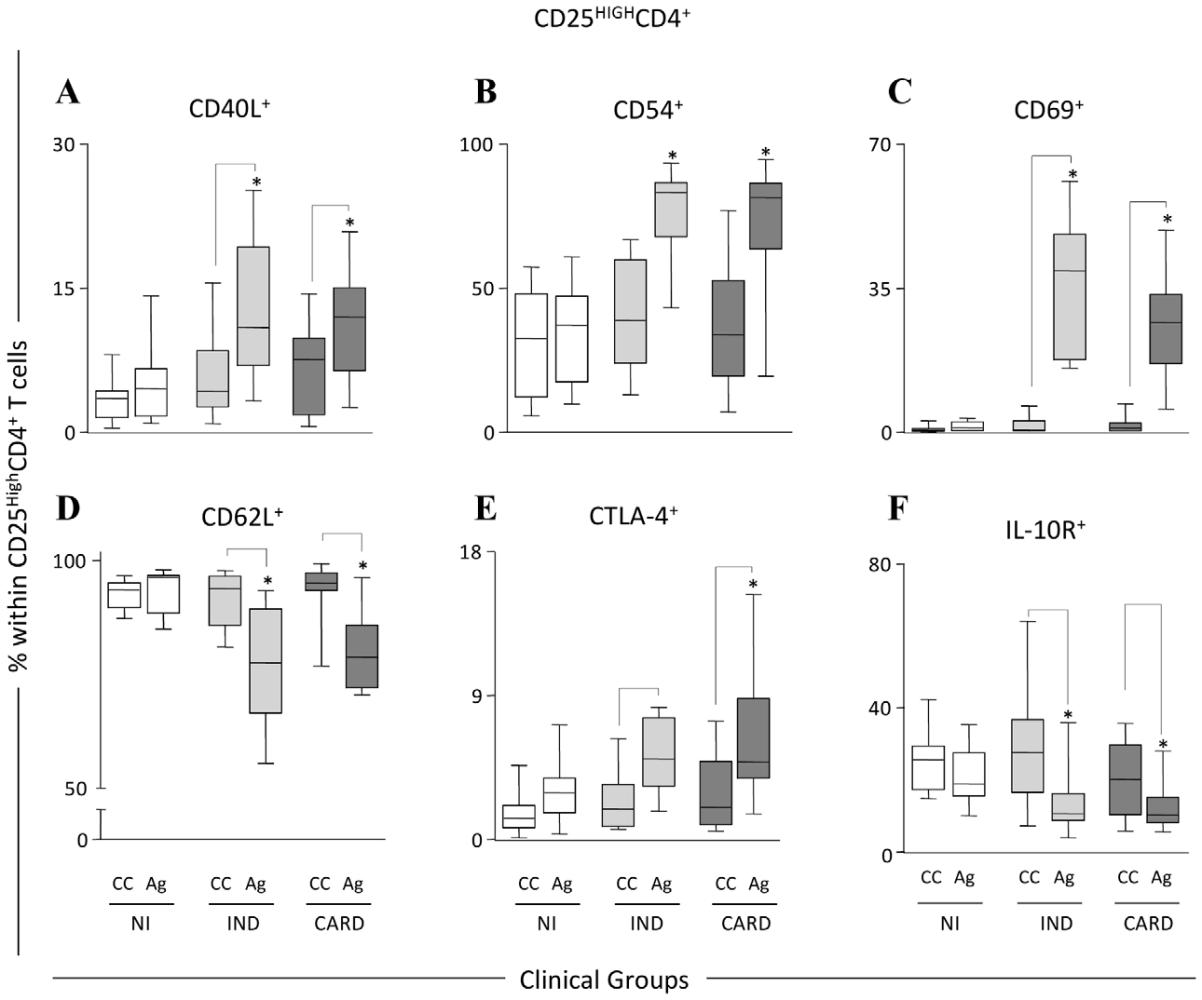
Phenotypic analysis of CD25^High^ CD4^+^ regulatory T cells in the peripheral blood from chagasic patients. Phenotypic features of CD25^High^ CD4^+^ cells from patients with distinct clinical forms of Chagas' disease (IND, light gray box; CARD, dark gray box) and non-infected individuals (NI, white box) following short-term in vitro stimulation of whole blood samples with *T. cr*u*zi* antigens. Baseline levels for a range of phenotypic features of CD25^High^ CD4^+^ cells were obtained from control cultures (CC) maintained under the same conditions (22 h incubation at 37°C, CO_2_ humidified incubator). The results are expressed in box plot format as the percentage of positive cells within CD25^High^ CD4^+^ cells including those expressing adhesion molecules CD62L (D) and CD54 (B), co-stimulatory receptors CD40L (A) and CTLA-4 (E), activation marker CD69 (C) and regulatory receptor IL-10R (F). The box stretches from the lower hinge (defined as the 25^th^ percentile) to the upper hinge (the 75^th^ percentile) and, therefore, contains the middle half of the score in the distribution. The median is shown as a line across the box. Therefore, 1:4 of the distribution is between this line and the bottom or the top of the box. Significant differences are identified by connecting lines for comparisons between CC and Ag, and highlighted that *T. cruzi* antigens triggered an overall change in the phenotypic features of CD25^High^ CD4^+^ cells towards lower frequency of CD62L^+^ and IL-10R^+^ cells besides increased levels of CD54^+^, CD40L^+^, and CD69^+^ cells in both IND and CARD groups. Although *T. cruzi* antigens were able to induce higher levels of CTLA-4 in both groups of chagasic patients (IND and CARD), the impact of *T. cruzi* antigens was more pronounced in CARD, leading to higher frequency of CTLA-4^+^ cells in comparison to NI. Adapted from [20].

With the objective of better illustrating the differences in this T cell population, we have further characterized the CD25^High^ CD4^+^ Treg cells after in vitro exposure of whole blood to *T. cruzi* antigens. As a result, it was observed that CD25^High^ CD4^+^ Treg cells of chagasic patients express a number of cell surface markers that have been associated with activation and migration. Human CD25^High^ CD4^+^ Treg cells from IND and CARD patients presented an increased expression of CD40L, CD54, and CD69 (Figure 2A, B, and C, respectively) and a decreased expression of CD62L (Figure 2D) in the peripheral blood. Baecher-Allan and colleagues [15] first suggested that the CD25^High^ CD4^+^ T cells may exist in a semi-activated state in vivo, expressing a number of surface antigens that are usually associated with activated T cells. Thus, Treg cells may be primed for an anamnestic response upon re-encountering the parasite antigens. Previous reports have shown increased levels of adhesion molecules (CD54 and LFA-1) on regulatory cell subsets [14], [44]–[46], suggesting that these cells are able to penetrate into inflamed tissues and may act directly at the sites of inflamation [16], [47]–[49]. Our results are in agreement with these findings and we suggest that in Chagas' disease CD25^High^ CD4^+^ T cells can migrate to the tissues and control the severe inflammatory response in individuals with the IND clinical form of the disease. Additional analyses are currently being undertaken by our group to further support this hypothesis.

**Figure 3:**
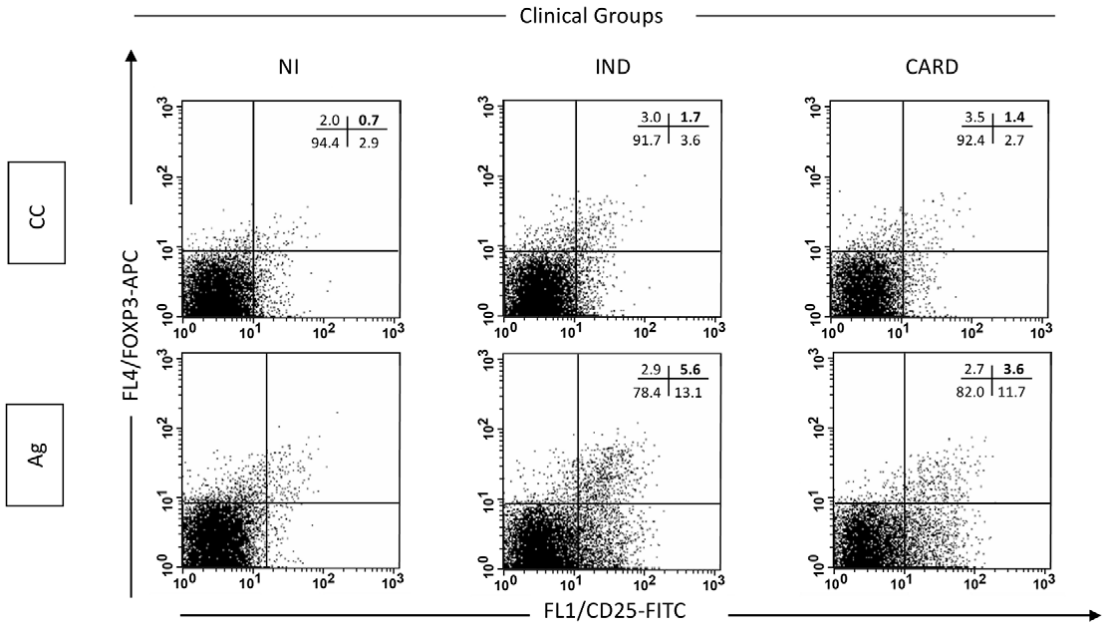
Analysis of Foxp3^+^ CD25^High^ CD4^+^ regulatory T cells in the peripheral blood from chagasic patients. Representative dot plots illustrate that the increased levels of regulatory T cells observed in *T. cruzi* antigens-stimulated cultures from IND tend to be higher than that observed in CARD, and also reflect an increased level of Foxp3^+^ CD25^+^ cells in the IND group (bottom graphs). Quadrant statistics were used for data analysis, and the results are expressed as the percentage of positive cells within the CD25^+^ CD4^+^ selected lymphocytes. Reproduced and adapted from [20].

In addition to the above shown cell surface markers, we evaluated the expression of Foxp3 by these cells. This marker has been shown to play a key role in Treg cell function and still represents a highly specific marker for these cells [50]–[54]. Previously published studies have consistently reported that Foxp3 is predominantly expressed by both human and murine CD25^High^ CD4^+^ Treg cells. In agreement with previous data, increased levels of Foxp3+ CD25+ cells were also observed in the IND group after in vitro stimulation with *T. cruzi* antigens (Figure 3) [20], emphasizing once again the role of Treg cells in the control of Chagas' disease morbidity.

Regulatory T cells are known to express CTLA-4 constitutively; however, the role played by this inhibitory signaling molecule in Treg cells homeostasis and in the regulation of the immune responses is not yet well defined. CTLA-4 expression has been associated with Treg cells production of immunosuppressive cytokines, such as TGF- β and IL-10, and is essential for cell-contact suppression in vitro [55], [56]. We observed a higher frequency of CTLA-4^+^ CD25^High^ CD4^+^ T cells in whole blood form CARD after in vitro stimulation with *T. cruzi* antigen, when compared with non-infected individuals (Figure 2E) [20]. Increased numbers of cIL-10^+^ cells (Figure 1) were observed in the CD25^High^ CD4^+^ T lymphocytes from both groups of chagasic patients (IND and CARD), suggesting that stimulation with *T. cruzi* antigens induces secretion of this cytokine by cells from individuals with the polar clinical forms of the disease. Preliminary data from our group show that while there is a positive correlation between cIL-10^+^ and CTLA-4^+^ cells in IND (spearman indexes *r* = 0.5985; *p* = 0.0008), the same was not observed in CARD (spearman indexes *r* = 0.3696; *p* = 0.0652).

An interesting study by Souza et al. 2007 [38] has shown that the expression of CTLA-4 in T cells from indeterminate patients was up-regulated; however, the expression of this molecule was not seen on the surface of cells from cardiac patients. Importantly, the same group observed higher frequency of IL-10 in IND patients after in vitro stimulation with *T. cruzi* antigens. The binding of CTLA-4 with anti–CTLA-4 mAbs or CD80 ligand was shown to inhibit IL-2 production and proliferation of primary CD4 T cells [57], [58] and IL-4 and IFN-gamma production of T-cell clones [59]. In addition, the inhibitory signal transduced by CTLA-4 binding is able to increase IL-10 and TGF-β, showing an additional effect in regulating this pathway [60], [61]. Therefore, CTLA-4 may exert its immunoregulatory role through a bidirectional signaling between CTLA-4 on T cells and B7 on APCs that result in attenuation of ongoing immune response and maintenance of T-cell homeostasis. CTLA-4 also acted indirectly through the development of regulatory T cells producing IL-10 [62], and the suppressive activity may require cell-cell contact and implies binding with CTLA-4 [55], [56]. Thus, the identification if the contact cell-cell through CTLA-4 to Treg cells produces IL-10 is crucial to understanding their role in Chagas' disease. These findings further support our hypothesis that the determining factor is a balance between cytokines and cell populations in these individuals. Additional analyses are currently being undertaken to further support this hypothesis.

**Figure 4:**
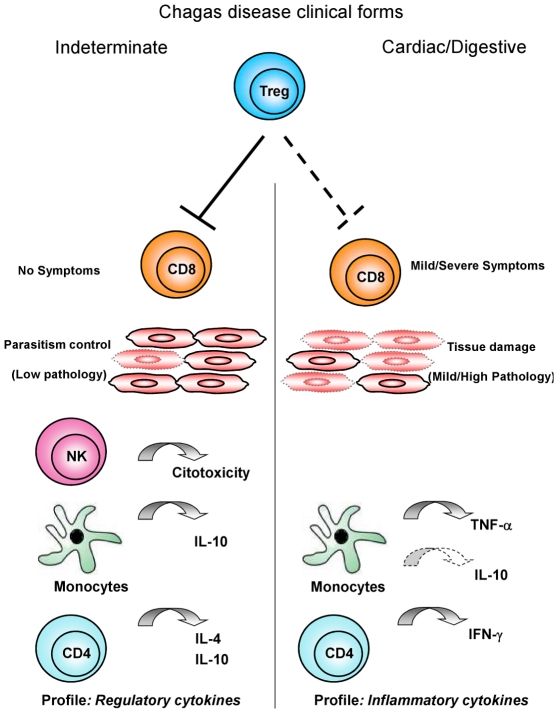
Proposed hypothesis for CD25^High^ CD4^+^ Treg cells function on immunoregulation in chronic Chagas' disease. Several leukocyte subsets have been shown to play a role in immunoregulation during chronic infections. In this model, Chagas' disease patients with the indeterminate clinical form show Treg cells able to modulate the effectors' function of CD8^+^ T cells, in a microenvironment supported by cytotoxic NK-cells, Monocytes and CD4^+^ T cells producing regulatory cytokines (IL-10 and IL-10, IL-4, respectively). This immunological milieu contributes to controlling the parasitemia and regulating the immunopathology. On the other hand, Chagas' disease patients with cardiac and digestive clinical forms display insufficient modulation by Treg cells with activated CD8^+^ T cells besides monocytes and CD4+ T cells producing inflammatory cytokines (TNF-α and IFN-γ, respectively). This microenvironment triggers immunopathological events and leads to tissue damage in the absence of regulatory mechanisms and cytotoxic NK-cell functions.

Other studies have shown that IL-10 is an important cytokine involved in the suppressive function of Treg/Tr1 cells [36], [63]. Although there is no clear evidence for the effector function of this cell population in Chagas' disease, we observed a high percentage of CD25^High^ CD4^+^ T cells from cardiac patients expressing CTLA-4, as well as an increase in the frequency of CD25^High^ CD4^+^ T cells expressing IL-10 (Figure 1) with a simultaneous decrease in the frequency of IL-10 receptor in IND and CARD (Figure 2F). Therefore, it is possible that CD25^High^ CD4^+^ Treg cells from IND patients may be important in controlling type 1 response to *T. cruzi*. Thus, a hypothesis to be further tested is that Treg cells, through expression of cIL-10, are beneficial to patients in the indeterminate clinical form by maintaining a balance between efficient effector cells that kill the parasites and avoiding the development of tissue immunopathology. On the other hand, in CARD patients, these cells are not sufficient and/or competent to control the inflammatory process mediated by high levels of activated CD8^+^ HLA-DR^+^ T cells as previously observed in the peripheral blood [10] and the inflammatory infiltrate of cardiac lesions [1], [64]. Thus, it is likely that CD25^High^ CD4^+^ T cells use different mechanisms to regulate the immune response during Chagas' disease and that the host-parasite interactions may be influenced by the ratio of regulatory/effectors T cells. Based on our observations, the model presented in Figure 4 may explain the putative mechanisms involved in the control and induction of pathology in Chagas' disease and is being used by our group as the test model of our hypotheses. We argue that the generation of protection or pathogenic responses in Chagas' disease is highly influenced by the suggested balance of this complex immune response induced by *T. cruzi* as previously shown by our group and others. Therefore, cardiac patients have specific cell populations involved in the establishment of an inflammatory cytokine profile and lack of control of the immune response. On the other hand, indeterminate patients have an effective, and possibly transitory, control of the response to the infection mediated by regulatory and effector T cells.

Moreover, we hypothesize that a higher cytotoxic activity of NK cell subsets in IND patients could be important in helping to suppress parasitemia to very low levels, resulting in avoidance of the development of a strong acquired immune response against parasite-specific antigens and the outcome of severe chagasic disease [65]. The ability to build up NK-mediated cell cytotoxicity seems to play a pivotal role in the generation of effective, nondeleterious inflammatory mechanisms. Considering the complexity of the human immune response, this insight suggests that other immunoregulatory mechanisms come into play to control the intense immune activity and are apparently necessary to prevent a deleterious effect of the excessive stimulation of the immune system, represented by CD25^High^ CD4^+^ Treg cells. Moreover, the ability of these cells to inhibit IFN-gamma synthesis confirms previous observations of low levels of IFN-gamma production by mononuclear cells from patients presenting with the asymptomatic chronic phase of Chagas' disease. In addition, the production of IL-10 by macrophages/monocytes comprises a supplementary regulatory mechanism to modulate the immune response in IND patients [3].

## Concluding Remarks

This review summarizes the most relevant results regarding the role of regulatory T cells in Chagas' disease. Although the available data do not cover all the gaps and did not address several questions regarding the mechanisms underlying the recruitment, activation, and function of regulatory T cells in development of tissue damage in Chagas' disease patients, they give important insights to clearly emphasize that Treg cells are relevant to modulate the immune response in chronic human *T. cruzi* infection (Box 3). Given the complex nature of the immune response in humans and the low availability of biological samples, the establishment of such protocols represents an important drawback in the human studies. Although the interpretation, suggestions, and conclusions presented here are based on phenotypic data (not on functional studies), they represent important advances toward the better understanding of the role that Treg cells may have in vivo in the different clinical forms of Chagas' disease. Moreover, it is important to highlight that while working with human diseases, it is difficult to perform a large number of functional studies, since functional analysis using human Treg cells requires a considerable amount of biological samples, in contrast with those facilities found when working with syngeneic experimental models.

**Box 3. Key Papers in the Field**

de Araújo FF, da Silveira AB, Correa-Oliveira R, Chaves AT, Adad SJ, et al. (2011) Characterization of the presence of Foxp3(+) T cells from patients with different clinical forms of Chagas' disease. Hum Pathol 42(2): 299–301.
Mariano FS, Gutierrez FR, Pavanelli WR, Milanezi CM, Cavassani KA, et al. (2008) The involvement of CD4^+^CD25^+^ T cells in the acute phase of *Trypanosoma cruzi* infection. Microbes Infect 10(7): 825–833.
Araujo FF, Gomes JA, Rocha MO, Williams-Blangero S, Pinheiro VM, et al. (2007) Potential role of CD4^+^CD25^HIGH^ regulatory T cells in morbidity in Chagas disease. Front Biosci 1(12): 2797–2806.
Vitelli-Avelar DM, Sathler-Avelar R, Massara RL, Borges JD, Lage PS, et al. (2006) Are increased frequency of macrophage-like and natural killer (NK) cells, together with high levels of NKT and CD4+CD25high T cells balancing activated CD8+ T cells, the key to control Chagas' disease morbidity? Clin Exp Immunol 145(1): 81–92.
Vitelli-Avelar DM, Sathler-Avelar R, Dias JC, Pascoal VP, Teixeira-Carvalho A, et al. (2005) Chagasic patients with indeterminate clinical form of the disease have high frequencies of circulating CD3+CD16-CD56+ natural killer T cells and CD4+CD25High regulatory T lymphocytes. Scand J Immunol 62(3): 297–308.

Once the relevant phenotypic features of Treg cells associated with distinct clinical forms of the disease are established, functional studies might be performed in pre-clinical investigations using in vitro or humanized experimental models. Understanding the mechanisms by which Treg cells are recruited and activated in Chagas' disease patients will contribute to the design of rational cell therapy approaches to balance protection/pathology and control clinical morbidity during the chronic disease onset.
